# Genotype–environment interaction in layer chickens in the growing stage: comparison of three genotypes at two different feeding levels with or without red mite (*Dermanyssus gallinae*) infestation

**DOI:** 10.5194/aab-64-447-2021

**Published:** 2021-10-15

**Authors:** Hakan Erdem, Türker Savaş

**Affiliations:** Department of Animal Science, Faculty of Agriculture, Çanakkale Onsekiz Mart University, 17020 Çanakkale, Turkey

## Abstract

This study investigated how early growth was affected in various chicken genotypes, which were fed ad libitum or restricted and with or without poultry red mite (PRM) infestation. Atak-S (AS), New Hampshire Red (NHR), and Light Sussex (LS) genotypes were used in the study. In total, 120 chicks were used from each genotype. Four groups were formed: feed-restricted (FR) and infested with parasite (P${}^{+}$), FR only, fed ad libitum and P${}^{+}$ , and fed ad libitum only. Feed restriction was applied as 20 % of the feed consumption of the group fed ad libitum the day before for each genotype. The study was conducted between 2 to 12 weeks of age. Weekly live weights and feed consumption were recorded, and the feed conversion ratio was calculated. Traps were placed in cages to count parasites. Regarding the live weight, NHR tolerated the PRM infestation in the ad libitum feed conditions better than other genotypes. While the infested NHR and AS birds had lower live weights than the non-infested ones under FR conditions, there was no difference between infested and non-infested birds of NHR and AS genotypes when they fed ad libitum. The feed consumption of infested AS and NHR birds was higher than that of non-infested counterparts when fed ad libitum. By contrast, the LS chicks consumed less food in the infested environment. In conclusion, the genotypes responded differently to PRM infestation in different feeding environments.

## 1 Introduction

Different genotypes in any environmental conditions can react differently to changing environmental conditions (Truberg and Huhn, 2000; Settar et al., 1999). This is called genotype and environment interaction (Bowman, 1972). Genotype–environment interaction (GE) can be seen as a change in the sequence of genotypes from one environment to another and as the difference in performance observed between environments or as a combination of the two (Truberg and Huhn, 2000). In GE, the performance of genotypes for a particular trait may increase/decrease in the same way, or one may increase and the other decrease, which is both biologically and economically important (Drinkwater and Hetzel, 1991). If GE is not significant, genetic performance can be determine with phenotypic means in different environments. However, if GE is significant, this mean will be masked by sub-environments where genotypes differ significantly in relative performance (Fox, et al., 1997). In other words, if there is a GE, the best genotype in any environment may not be the best genotype for the same phenotype in another environment (Mulder and Bijma, 2005).

The existence of GE can reduce the efficiency of breeding programs (Hammami et al., 2009). For this reason, GE is significant for the efficiency and sustainability of breeding programs in order to have information regarding which genotype has the best and worst performance in which environment.

Studies of GE have generally been conducted in environments that differ in one factor or are cumulative environments. How GE performs in interactions between environments is not well known. It is even more difficult to explain GE in nested environments. In such conditions, it is necessary to approach the problem by estimating the qualities of the environments that affect performance. In other words, the hierarchy of the environments must be determined.

In this study, two environments with intertwined factors (parasite and feeding level), whose qualities are sharply separated from each other, are the subject as part of GE. In terms of the parasite environment, an environment contaminated with poultry red mite (*Dermanyssus gallinae*) is considered (Kirkwood,1967; Chauve, 1998, Sleeckx et al., 2019; Erdem et al., 2020; Yazgan et al., 2020). When birds are infested with poultry red mite (PRM), the organism primarily tries to increase the blood cell count to compensate for the loss of blood (Kilpinen et al., 2005). In addition, birds must overcome itching and skin irritation, which is a direct effect of PRM (Kilpinen et al.,2005; Kaoud and El-Dahshan, 2010; Sparagano et al., 2014; Erdem et al., 2020). Discomfort due to parasite infestation reduces the welfare of birds and decreases immunity; thus, it may cause growth retardation in young birds (Konyalı et al., 2013; Erdem et al., 2020). Qualitative and quantitative feed restriction, especially in broiler breeding, is widely used to control the effects of rapid growth (Hocking et al., 2002; Sahraei, 2012). The feeding level was chosen as a second environmental factor in the study because live weight gain, feed conservation ratio, and body condition deteriorate because of protein, energy, or feed intake restriction in animals (Yu et al., 1990; Roberts et al., 2007). On the other hand, if feed consumption is restricted in animals, it minimizes the basal metabolism, and growth and development slow down (Pym and Dillon, 1973; Yambayamba et al., 1996; Hornick et al., 2000).

Although Konyalı (2016) reported that the growth and development of different laying chicken genotypes were similarly affected by PRM infestation, it is not known how the interactions of different environments affect genotypes. There are indications that dietary factors may affect disease resistance (Blazer, 1992) and influence the severity of parasite infestations (Gyorgy, 1938; Ely and Harvey, 1969). On the other hand, it is not known whether restricted feeding in infestation conditions with PRM affects genotypes differently. In the light of these facts, the hypothesis of this study is that growth and development will be similarly affected by feed restriction of genotypes infested with PRM. In addition, it was accepted that the feeding level hierarchically represented the “upper environment” compared to the PRM infestation. This study therefore investigated how growth was affected in different laying chicken genotypes which were in the early growing stage and which were infested with PRM under feed restriction, and it examined whether genotypes responded equally to feed restrictions ins infested and non-infested environments.

## 2 Materials and methods

This study was conducted within the scope of the PhD thesis entitled “Quantitative Genetic Studies on Growth: Genotype Environment Interaction, Inbreeding and the Uniformity Problem” (unpublished). The research protocol was approved by the Ethics Committee of Çanakkale Onsekiz Mart University (approval date and number: 23 February 2018 – 2018/02-03).

In total, 120 female chicks were used from each genotype: Atak-S (AS), New Hampshire Red (NHR), and Light Sussex (LS). AS is a layer hybrid based on breeds of Rhode Island Red and Barred Rock developed at the Ankara Poultry Research Institute. The NHR genotype was developed in the US through intensive selection for the egg yield from Rhode Island Red. LS is a breed derived from local genotypes in the county of Sussex, United Kingdom.

The chicks were placed in cages at the age of 2 weeks with four chicks per cage. Two environmental factors known to affect each other, namely feeding level (F) and PRM infestation (P), were considered. In this sense, four groups were created from each genotype (feed-restricted and infested (FR / P${}^{+}$), only feed-restricted (FR/P${}^{-}$), fed ad libitum and infested (AL / P${}^{+}$), and only fed ad libitum (AL / P${}^{-}$)). The feed-restricted groups consisted of eight cages, and the groups fed ad libitum consisted of seven cages for each genotype. Feed restriction was applied as 20 % of the 1 d feed consumption for the AL / P${}^{-}$ group of each genotype.

Birds were fed with a diet containing 21 % crude protein up to 8 weeks of age and 15 % CP feed was used for the next 4 weeks. During the study, a 16L : 8D photoperiod was applied. Weekly live weight (LW) and feed consumption were observed with a 0.05 g precision scale. In the ad libitum groups, daily feed intake (DFI) per animal was recorded. The feed conversion ratio (FCR) per animal in both ad libitum and feed-restricted groups was calculated (FCR: (feed, g) ⋅ (live weight gain, g)${}^{-1}$).

*D. gallinae* were collected from backyard henhouses from in and around Çanakkale city. Almost equal amounts of mites were placed in each trap/cage. The parasite population dynamic was observed in the traps. The traps were photographed at 6, 8, 10, and 12 weeks of age of the birds. The average number of adult mites per 1 cm${}^{2}$ in a trap was counted; the mite population size was estimated. The area covered by the mite population was determined in the photographs, and the mite load was estimated. The study was terminated at the age of 12 week.

### Statistical analysis

Analyses were completed separately for each FR and AL group. The variance analysis method was used in the model that includes genotype, parasite, and their interaction in the analyses of the initial and end-of-study live weights (Eq. 1).

1 $$Y_{ijk}=\mu +g_{i}+p_{j}+gp_{ij}+e_{ijk},$$

where $Y_{ijk}$ is the initial live weight or the end-of-study live weight, μ is the population mean, $g_{i}$ is the fixed effect of the ith genotype, $p_{j}$is the fixed effect of the jth parasite group, $gp_{ij}$ is the interaction of genotype and parasite, and $e_{ijk}$ is the random residual.

The repeated measure variance analysis method was used in the model that includes genotype, parasite, age (week), and their interaction in the analysis of live weight by weeks (Eq. 2). In this model, the initial body weight (IBW) was included as a covariate. In the statistical analysis of DFI and FCR, the same model and method were used, except for the initial live weight (covariate). As a result of these analyses, interactions that were not significant were removed from the model.

2 $$\begin{array}{rl} Y_{ijklmo} & =\mu +c_{ijklm}+g_{j}+p_{k}+w_{l}+\beta x_{ijklm}\\ & +(gXp{)}_{jk}+(gXw{)}_{jl}+(pXw{)}_{kl}\\ & +(gXpXw{)}_{jkl}+e_{ijklmo}, \end{array}$$

where $Y_{ijklmo}$ is the weekly live weight, μ is the population mean, $c_{ijklm}$ is the random effect of the mth chick within jth genotype, kth parasite group, and age of lth week, $g_{j}$ is the fixed effect of the jth genotype, $p_{k}$ is the fixed effect of the kth parasite group, $w_{l}$is the fixed effect of the wth week, β is the regression coefficient, $x_{ijklm}$ is the initial live weight of the mth chick of the ith genotype and the jth parasite group, and $e_{ijklmo}$ is the random residual.

The regression coefficients were used to compare the slopes of the weekly live weights of all subgroups. Orthogonal contrasts derived from the general linear model with an age–parasite–feeding–genotype interaction effect on weekly live weights were used to compare regression coefficients associated with each subgroup.

All the analyses were carried out with the SAS package program (2002).

## 3 Results

In Table 1, the least square means of live weight at the beginning and end of the study are shown for genotype, F, and P effects. As expected, there are significant differences between genotypes in terms of live weight at the beginning and end of the study (P=0.0122 and P<0.0001). No significant effect was found for the parasite–genotype interaction (PG) (P=0.2399).

However, P affected the live weight of the birds in the restricted-feeding conditions at the end of the study (P=0.0058). There is also a difference between genotypes in terms of live weight at the end of the trial (P<0.0001).

**Table 1:** Least square means ($\bar{x}$) of live weights at the beginning and end of the study and their standard errors (SEs) and significance levels (P) under ad libitum and restricted-feeding conditions in infested (P${}^{+}$) and non-infested (P${}^{-}$) birds.

	Ad libitum				Restricted			
	Initial of study		End of study		Initial of study		End of study	
	$\bar{x}$	SE	$\bar{x}$	SE	$\bar{x}$	SE	$\bar{x}$	SE
AS	88.06	1.947	969.18	20.440	86.35	1.710	843.34	15.770
NHR	93.87	1.965	1242.70	20.963	93.31	1.710	1006.40	16.085
LS	85.79	1.947	1032.22	20.612	86.99	1.710	907.44	15.729
P value								
Parasite	0.9684		0.6962		0.9395		0.0058	
Genotype	0.0122		<0.0001		0.0072		<0.0001	
PG	0.9750		0.2399		0.5463		0.8432	

**Figure 1:**
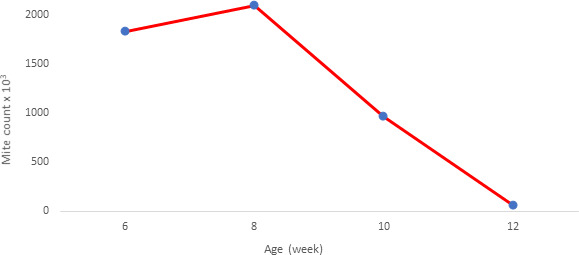
Mite population by weeks.

The mite population was monitored from the age of 6 weeks of the chicks (Fig. 1). A decrease was observed after 8 weeks of age when the mite population was at the highest number with approximately 2 million.

**Table 2:** Significance levels of variation sources for weekly live weights (LWs, g), feed conversion ratios (FCRs) separated by ad libitum and feed-restricted birds, and daily feed intake (DFI) per bird for birds fed ad libitum.

	LW		FCR		DFI
Source of variation	AL	FR	AL	FR	AL
Initial live weight	<0.0001	<0.0001	–	–	–
Age	<0.0001	<0.0001	<0.0001	<0.0001	<0.0001
Parasite	<0.0001	<0.0001	0.6743	0.0481	0.0417
Genotype	<0.0001	<0.0001	<0.0001	0.0015	<0.0001
Age–parasite	0.2129	<0.0001	<0.0001	<0.0001	0.0002
Age–genotype	<0.0001	<0.0001	0.0110	0.0500	0.1016
Parasite–genotype	<0.0001	0.1309	0.2238	0.6802	0.0004
Age–parasite–genotype	0.9966	1.0000	0.7497	0.0005	0.8988

**Figure 2:**
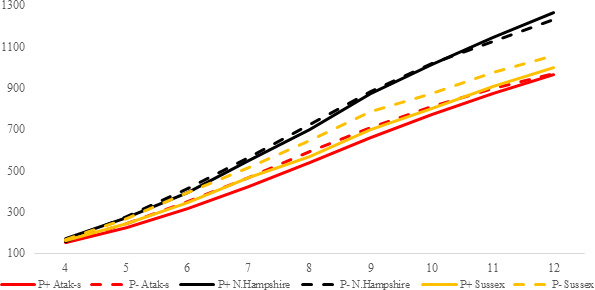
Trends of weekly average live weights for P${}^{+}$ and P${}^{-}$ groups by genotype in an ad libitum environment (grams).

**Figure 3:**
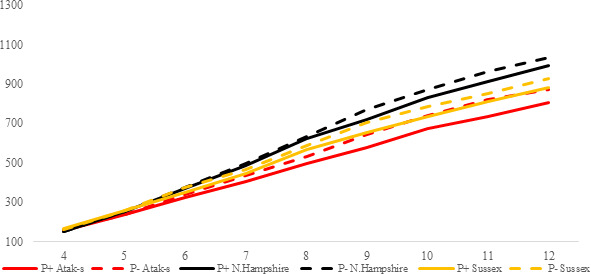
Trends of weekly average live weights for P${}^{+}$ and P${}^{-}$ groups by genotype in feed-restricted environment (grams).

The P values of the variation sources related to weekly average live weight for parasite and genotype subgroups are shown in Table 2 for each feeding environment. Their trends are seen in Figs. 2 and 3. In AL, the weekly live weights were significantly influenced by parasite, genotype, and PG effects (P<0.0001). The NHR genotype had the highest live weight means during the study both in P${}^{+}$ and P${}^{-}$. The NHR tolerated the PRM infestation in AL conditions and performed similarly based on weekly live weights in both P${}^{+}$ and P${}^{-}$. At the end of the study, AL birds both with or without parasite infestation had similar live weights. However, the infested birds in the LS genotype during the study under AL feeding conditions had a lower LW average than the non-infested birds.

In the FR conditions, the difference is significant in weekly average live weight both in parasite environments and between genotypes (P<0.0001). Especially after the age of 8 weeks, the difference in live weight between the non-infested and infested groups is remarkable. Under restricted-feeding conditions, the AS birds were more affected by the infestation than the other genotypes. The PG was not significant (P=0.1309).

**Table 3:** Regression coefficients (b, g) and their standard errors (SEs) showing the slopes of the weekly live weights according to feeding level and parasite effects.

Feeding	Parasite	Genotype	b	SE
	Infested (P${}^{+}$)	Atak-S	12.28${}^{\text{a}}$	0.097
		New Hampshire Red	16.35${}^{\text{b}}$	0.224
		Light Sussex	12.71${}^{\text{c}}$	0.125
Ad libitum	Non-infested (P${}^{-}$)	Atak-S	12.81${}^{\text{c}}$	0.098
		New Hampshire Red	16.18${}^{\text{b}}$	0.245
		Light Sussex	13.88${}^{\text{d}}$	0.109
	Infested (P${}^{+}$)	Atak-S	10.61${}^{\text{e}}$	0.073
		New Hampshire Red	13.72${}^{\text{d}}$	0.196
		Light Sussex	11.68${}^{\text{f}}$	0.072
Restricted	Non-infested (P${}^{-}$)	Atak-S	11.57${}^{\text{f}}$	0.075
		New Hampshire Red	14.05${}^{\text{d}}$	0.175
		Light Sussex	12.54${}^{\text{ac}}$	0.102

All the regression coefficients are significantly different from 0 (P<0.0001). Different letters indicate significant differences between the regression coefficients (P<0.05).

In the feeding-level–parasite–genotype subgroups, the NHR is the fastest-growing genotype in both AL and FR environments in terms of the regression coefficients of weekly (age) live weights (Table 3). These birds also grow equally in P${}^{+}$ and P${}^{-}$ environments in both feeding environments. The growth rate of the AS birds in P${}^{+}$is relatively lower (4 %) than that of the P${}^{-}$ birds. The LS genotype shows the same situation where the growth rate is 8 % lower. P${}^{-}$ birds show a better growth performance than P${}^{+}$ birds in AS and LS genotypes.

In the FR conditions, the NHR genotype is again the fastest-growing genotype. Also, the growth rate of the P${}^{+}$ and P${}^{-}$ birds is close in these birds. In the AS genotype, the P${}^{+}$ birds have an 8 % lower slope than the P${}^{-}$ birds. In the LS genotype, the P${}^{+}$ birds have a 7 % lower slope than the P${}^{-}$ birds. As in the AL conditions, the P${}^{-}$ birds of the AS and LS genotypes also have a better growth performance than the P${}^{+}$ birds in the FR conditions. In the LS genotype, the proportional difference in growth rate between the AL / P${}^{+}$ and FR / P${}^{-}$ environments is close. On this basis, it can be said that, for the LS genotype, the infestation has almost the same effects in both feeding environments. In the AS genotype, however, the infected birds show a growth rate that is twice as low in the FR environment as it is in the AL environment.

**Figure 4:**
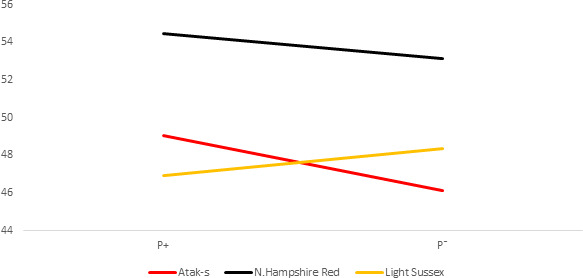
Daily feed intake (grams) per bird (DFI) for infested (P${}^{+}$) and non-infested (P${}^{-}$) groups in ad libitum conditions (AL).

The P values of the sources of variation regarding the tendency of the DFI averages of the genotype and parasite subgroups on the basis of weekly ages in the AL environment are shown in Table 2, whereas their averages are shown in Fig. 4. Parasite environment, genotype, and their interactions are significant for DFI (respectively, P=0.0417, P<0.0001, P=0.0004). The AS and NHR birds grown in the infested environment consumed more feed than those grown in the non-infested environment. By contrast, the LS genotype consumed less feed in the infested environment than in the non-infested. According to the feed restriction method of the study, the feed intake of the FR groups was determined by the AL / P${}^{-}$ groups. Therefore, the FR / P${}^{+}$ and FR / P${}^{-}$ birds consumed 20 % less feed than the AL / P${}^{-}$ groups.

**Figure 5:**
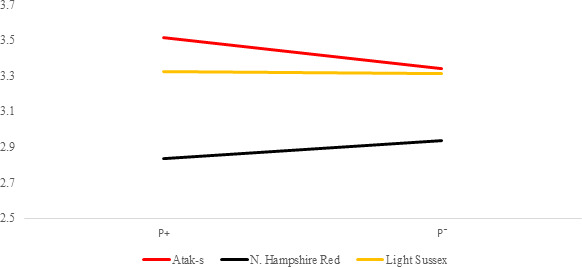
Averages of feed conversion ratio for infested (P${}^{+}$) and non-infested (P${}^{-}$) groups in ad libitum conditions (AL); feed g / LW g.

**Figure 6:**
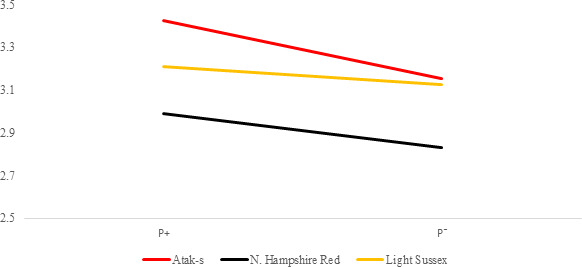
Averages of feed conversion ratio for infested (P${}^{+}$) and non-infested (P${}^{-}$) groups in feed-restricted conditions (FR); feed g / LW g.

Table 2 shows the P values of the sources of the variation in the FCR at weekly age in the genotype and parasite subgroups in the AL and FR environment. Its averages are shown in Figs. 5 and 6. There is a difference between the genotypes in terms of FCR in the AL environment (P<0.0001). While NHR is the best genotype in terms of FCR, AS and LS are similar. Genotype–parasite interaction is not significant (P=0.2238). The best FCR is found for the NHR genotype in the P${}^{+}$ group, while the average FCR has nearly the same value in the AS and LS genotypes in the P${}^{-}$ groups. The FCR value of the birds of the AS genotype decreased from 3.52 to 3.34, while that of the birds of the NHR increased from 2.83 to 2.93 in the P${}^{+}$ and P${}^{-}$ groups, respectively. In the LS genotype, this value almost did not change (P${}^{+}=3.32$, P${}^{-}=3.31$).

In the FR environment, genotype and parasite effects influenced FCR significantly (P=0.0015, P=0.0481, respectively). However, the genotype–parasite interaction was not significant (P=0.6802). The mean FCR of the P${}^{-}$ groups had almost the same values in the AS and LS genotypes in the FR environment as in the AL environment. In the AS and LS genotypes, the mean FCR values decreased from the P${}^{+}$ environment to the P${}^{-}$ environment (from 3.42 to 3.15 and from 3.21 to 3.12, respectively). Different from the AL environment, the mean FCR values of the NHR genotype in the FR environment decreased from the P${}^{+}$ environment to the P${}^{-}$ environment, in the same direction as for the other genotypes (from 2.99 to 2.83). Considering that the effect of the parasite is important in the AL environment and is not important in the FR environment, this situation points to an interaction in terms of environments related to FCR in the NHR genotype.

## 4 Discussion

While the study shows a significant PG interaction associated with LW in the birds fed ad libitum, it is not significant for the feed-restricted birds. The genotypes were affected differently by infestation in the AL environment but were similarly affected by infestation in the FR environment. The PG interaction related to FCR was not seen in both feeding environments. The PG interaction occurred in the birds in terms of DFI, which, of course, led to variation only in the AL environment.

Feed restriction is frequently used in poultry breeding, especially in broiler breeding, in order to prevent metabolic disorders and to keep live weight under control (Zubair and Leeson, 1994; Balog et al., 2000; Urdaneta–Rincon and Leeson, 2002; Camacho et al., 2004; Fassbinder–Orth and Karasov, 2006). However, the lack of nutrients (protein, energy, amount, etc.) during the growth phase leads to negative effects on growth such as decrease in live weight gain and deterioration in FCR (Vaughters et al., 1987; Plavnik and Hurwitz, 1990; Acar et al., 1995). The organism may have difficulties in dividing the insufficient supply of nutrients between internal complex functions (such as growth, development, repair, defense, and reproduction) (Coop and Kyriazakis, 1999). This situation may cause a malfunction in some body functions. On the other hand, some authors report that feed restriction does not affect immune-related parameters and that it, on the contrary, increases innate immunity (Hangalapura et al., 2005; Fassbinder-Orth and Karasov, 2006; Klasing, 2007; Khajavi et al., 2010).

In the AL environment, the LW difference between the P${}^{+}$ and P${}^{-}$ birds' decrease or is not observed at all when the NHR and AS genotypes are considered (Fig. 2). The higher feed consumption of the P${}^{+}$ birds compared to the P${}^{-}$ birds (Fig. 4) may lead to overcoming the negative effects of the infestation. However, the opposite is the case with the LS genotype. The birds infected with PRM have a lower feed intake and a lower live weight than the non-infested LS. In fact, there are contradictory reports regarding the fact that the mite infestation causes birds to reduce or increase their feed consumption (Williams, 2003; Mul et al., 2009; Erdem et al., 2020). While the NHR and LS genotypes used in the study are pure breeds, the AS genotype is a hybrid one. However, this does not explain why NHR and AS have a higher feed intake, whereas LS has a lower feed intake. Hybrid genotypes are expected to be more resistant to bad environmental conditions than pure genotypes due to the heterosis effect (Ali et al., 2000). Indeed, when the infested and non-infested chicks are compared, the results in the ad libitum environment show relatively better growth for the infested AS birds versus the LS birds. However, this result is not supported in feed-restricted birds.

Since the LW means of the genotypes are almost parallel to one another, Fig. 3 confirms that there is no interaction between genotype and environment in the FR conditions (P=0.1309). In the FR environment, it can be clearly seen that the P${}^{+}$ birds have a lower LW than the P${}^{-}$ birds, even though the groups received the same amount of food.

When the growth of the AS and NHR genotypes is considered, it appears that a negative effect (the PRM infestation) is mitigated by a positive effect (ad libitum feeding). However, growth will be adversely affected if the appropriate conditions are not maintained (restricted feeding). Accordingly, the growth of the birds in the FR environment was significantly slowed compared to the AL birds and the NHR birds with the highest live weights were most affected. The AS and LS chicks were similarly affected by the feed restriction.

According to Shelford's law of tolerance (1931), an organism can survive in a range of lower and upper limits of a factor (tolerance range); in other words, it can tolerate this factor in this range. The tolerance range may vary depending on factors, as well as on organisms in terms of the same factor. Our results suggest that the live weight differences for each genotype between the infected birds and non-infested birds are smaller in the AL environment than in the FR environment. According to these results, the feeding environment is a limiting factor in the impact of the infestation.

The DFI means of the genotypes in Fig. 4 show a clear GE, resulting in a change in the genotype ordering (crossover). While the LS chicks consumed less food in the infested group than in the non-infested group, the AS and NHR birds consumed more in the infested group than in the non-infested group. However, while the differences of FCR between the P groups in both feeding environments are not significant, the apparent GE in Figs. 5 and 6 is also not significant. On the other hand, when looking at Figs. 5 and 6, there are slight differences in the slope of the genotypes due to the significant effects of the PRM in the FR environment compared to the insignificance in the AL environment. It is reported that parasitic diseases have adverse effects on FCR (Phengvichith and Ledin, 2007; Tellez et al., 2014; Yin et al., 2014). It is known that *Dermanyssus gallinae* increases feed intake and decreases growth, whereas it causes a deterioration in FCR (Kirkwood, 1967; Williams, 2003; Sleeckx et al., 2019; Erdem et al., 2020). In contrast, Erdem et al. (2020) reported that PRM-infested quails consume less food than non-infested quails. The direct or indirect effects of the parasites can lead to the underutilization of the nutrients used for growth and development, which worsens homeostasis. To maintain homeostasis, the host can increase feed intake, which could close the energy and protein deficit. However, if the host is unable to cope with the stress caused by the parasite, feed consumption may be reduced. Considering DFI and FCR, the genotypes appear to have different responses to infestation even when exposed to the same feeding environment.

In the FR environment, the NHR genotype appears to have difficulties in evaluating the feed offered in the P${}^{+}$ group for growth and development as opposed to the P${}^{-}$ group. It probably uses up some of its energy fighting the parasite. The NHR and AS chicks in the AL environment were not affected by this adverse situation by probably increasing feed intake. However, the effects of infestation are likely to be different for the LS genotype than for the other genotypes. Different mechanisms may underlie the reduction in feed intake under the PRM infestation. One of these may be the increase in scratching frequency caused by mite bites, which reduces the time the animal takes for feed intake (Konyalı et al., 2018; Erdem et al., 2020). Another reason can be called anorexia since the parasite causes physiological discomfort. It has been reported that brain serotonin activity, known to affect appetite physiology, is affected by external parasite infestations (Øverli et al., 2014).

## 5 Conclusions

A clear result of this study is that the change in the feeding environment caused changes in the reactions of the genotypes to the PRM infestation. Therefore, our hypothesis regarding the fact that the growth of the genotypes behaves similarly at different feeding levels in combination with the challenge of PRM infestation or non-infestation was rejected. However, our assumption that the feeding environment is hierarchically above the parasite infestation was proven.

Our results showed that a bird's genotype and feeding environment affect responses of young layer chickens to parasite challenge, and the results also indicated significant GE for growth performance.

## Data Availability

The data are available from the corresponding author upon request.
